# Editorial: Social capital and wellbeing of teachers and principals: Social support and beyond

**DOI:** 10.3389/fpsyg.2022.1127740

**Published:** 2023-01-09

**Authors:** Simon Beausaert, Eva Kyndt

**Affiliations:** ^1^Department for Educational Research and Development, Maastricht University, Maastricht, Netherlands; ^2^Centre for Transformative Innovation, Swinburne University of Technology, Hawthorn, VIC, Australia

**Keywords:** social capital, wellbeing, teacher, principal, social support

Teachers and principals are increasingly required to fulfill multiple roles (i.e., teacher, coach, manager, administrator, etc.) as societal expectations for education keep growing. In addition, the demand for accountability (i.e., through centralized exams, etc.) keeps growing (Phillips and Sen, 2011). As job demands increase, teachers and principals are also increasingly dependent on decisions of central administrators—decisions they do not always agree with—which might give them the feeling they are not in control. The combination of increased demands and reduced decision latitude is putting their wellbeing at risk (Schaufeli and Taris, 2014).

The threat to teachers and principals' wellbeing is worrisome in itself but also likely to have consequences for effective teaching and student learning. Prior research has argued that principals are one of the key elements contributing to teacher success and, which are in turn the main driver of student achievement (Leithwood et al., 2012; Ni et al., 2018). Fortunately, the wellbeing of both principals and teachers is getting more attention from policy makers and broader educational leadership (Sahlberg, 2015).

There is a large body of research on teacher wellbeing. However, prior research is skewed toward individual attributes and contextual factors, often disregarding the complex nature of individuals being embedded in networks of interrelationships. These networks provide both affordances and constraints, which are the breeding ground for social contagion processes, which can foster positive or negative spirals (Meredith et al., 2020).

In response, this Research Topic focuses on the role of social networks and social capital for teacher wellbeing. Social capital refers to “the actual and potential resources embedded in relationships among actors” (Leana and Pil, 2006, p. 353). In general, different forms of social capital have been distinguished, based on the type of relationships formed, namely internal or external to the organization or school, or between people with the same or different formal power and/or authority. These relationships know a certain strength and might involve instrumental of personal relationships, which are more or less frequent and have various levels of trust embedded in them (Nahapiet and Ghoshal, 1998).

There seems to be a growing consensus that the social capital of teachers and principals might buffer against the high demands put on them and thus promote their wellbeing (Bauer et al., 2019). Although social support for teachers and principals and its relationship with their wellbeing has been studied a lot (e.g., Greenglass et al., 2020), social capital goes further than feeling socially supported. Recent research has for example shown that burnout is contagious through a variety of social interactions (Kalish et al., 2015; Meredith et al., 2020). This network effect was demonstrated even after controlling for traditional predictors such as workload and autonomy (Bakker et al., 2005).

This Research Topic aims to provide the reader an understanding of the complexity, depth, and variety in the relatively nascent research field of social capital for teacher and principal wellbeing. As such, it comprises articles with different perspectives on social capital, different operationalization's of wellbeing, different levels of education, and different methodologies.

In terms of the different perspectives on social capital, Coppe et al. conceptually clarified the meaning of the term social capital, keeping in mind previous teacher research, arguing for making a distinction between individual and collective social capital. The different empirical operationalization's that were used included social support for learning through learning communities (Gast et al.), manager support (Pöysä et al.; Gearhart et al.), job support (Li et al.), climate and administrative support, and facilities, general social support (Froehlich et al.), brokerage roles (Rechsteiner et al.), or the network structure of interpersonal relationships (Aboutalebi Karkavandi et al.).

Regarding wellbeing, the majority of selected studies focus directly on wellbeing using measures such as general wellbeing (Panadero et al.), work engagement (Li et al.), job stress (Pöysä et al.), emotional exhaustion, and burnout (Aboutalebi Karkavandi et al.). Other focused on interventions designed to support teachers' wellbeing such as teachers' engagement in formal and informal stress management interventions (Gearhart et al.), undertaking professional development activities for occupational wellbeing (Gast et al.) or social support interventions for newly qualified teachers (Froehlich et al.). In addition, within the selection of articles, the studies of Panadero et al., Pöysä et al., and Froehlich et al. paid specific attention to the role of emergency remote teaching during the COVID-19 pandemic.

Furthermore, this Research Topic demonstrates the variety in methodological approaches in the field. Quantitative (e.g., Rechsteiner et al.), qualitative (e.g., Gast et al.), and mixed methods (e.g., Froehlich et al.) all contribute to understanding this complex Research Topic. We want to highlight the studies of Aboutalebi Karkavandi et al. and Rechsteiner et al. who both collected social network data. Aboutalebi Karkavandi et al. specifically focused on the relation between different types and structures of interpersonal networks and burnout. Rechsteiner et al. specifically studied the role of being in a brokerage position, the degree to which a person occupies a bridging position between disconnected others, for wellbeing.

Finally, as mentioned, the teachers and principals in the samples under study were working across various levels of education: kindergarten (e.g., Li et al.), primary schools (e.g., Rechsteiner et al.), secondary schools (e.g., Aboutalebi Karkavandi et al.), and higher education (e.g., Gast et al.).

## Author contributions

SB and EK: writing and editing. Both authors contributed to the article and approved the submitted version.
